# TGF-Beta Signaling in Bone with Chronic Kidney Disease

**DOI:** 10.3390/ijms19082352

**Published:** 2018-08-10

**Authors:** Yoshiko Iwasaki, Hideyuki Yamato, Masafumi Fukagawa

**Affiliations:** 1 Department of Health Sciences, Oita University of Nursing and Health Sciences, Oita 870-1163, Japan; iwasaki@oita-nhs.ac.jp; 2 Division of Nephrology and Metabolism, Tokai University School of Medicine, Kanagawa 259-119, Japan; hyamato0602@yahoo.co.jp

**Keywords:** bone remodeling, mineral metabolism disturbance, chronic kidney disease, humoral factors

## Abstract

Transforming growth factor (TGF)-β signaling is not only important in skeletal development, but also essential in bone remodeling in adult bone. The bone remodeling process involves integrated cell activities induced by multiple stimuli to balance bone resorption and bone formation. TGF-β plays a role in bone remodeling by coordinating cell activities to maintain bone homeostasis. However, mineral metabolism disturbance in chronic kidney disease (CKD) results in abnormal bone remodeling, which leads to ectopic calcification in CKD. High circulating levels of humoral factors such as parathyroid hormone, fibroblast growth factor 23, and Wnt inhibitors modulate bone remodeling in CKD. Several reports have revealed that TGF-β is involved in the production and functions of these factors in bone. TGF-β may act as a factor that mediates abnormal bone remodeling in CKD.

## 1. Introduction

Transforming growth factor (TGF)-β superfamily molecules play critical roles in tissue development and differentiation in an autocrine/paracrine/endocrine manner [1]. In bone also, TGF-β family members are involved in the control of cell activities and metabolism throughout osteogenesis [2,3]. Intramembranous ossification and endochondral ossification are skeletal ossification processes that take place following condensation of mesenchymal stem cells in fetal skeletal development [4,5]. Intramembranous ossification occurs in some parts of the clavicles, neurocranium, and viscerocranium. During intramembranous ossification, mesenchymal stem cells differentiate directly into osteoblasts. Endochondral ossification takes place in the skull base and the posterior part of the skull, the axial skeleton, and the appendicular skeleton. During the endochondral ossification process, mesenchymal cells undergo chondrogenesis to form cartilage, which is later replaced by mineralized bone. TGF-β controls these processes. For example, TGF-β1-deficient mice display reduced bone growth and mineralization [6]. TGF-β2^−/−^ and TGF-β3^−/−^ double knockout mice display a lack of distal parts of the rib [7]. Especially, TGF-β2-null mice display severe skeletal abnormalities in both intramembranous and endochondral ossification [8].

Apart from bone development, TGF-β and its signaling are important for adult bone remodeling [9,10]. The skeleton in adults undergoes continuous renewal throughout life to maintain bone mass and bone strength to resist fracture. This process is critically dependent on the activities of two cell types and their interactions. Osteoclasts, which are derived from hematopoietic cells, resorb old bone matrix. Osteoblasts, which are derived from mesenchymal cells, deposit new bone matrix in the resorbed area and facilitate mineralization. TGF-β is intimately involved in each stage of these processes. TGF-β regulates recruitment of osteoclasts and osteoblasts, the crosstalk mediating bone remodeling, and the quality of bone matrix. Disturbance of the balance between bone resorption and bone formation underlies osteoporosis, which is characterized by reduced bone mass and deterioration of bone microarchitecture, resulting in increased fragility and fracture risk. In this review, we focus on the effect of TGF-β on bone remodeling and discuss the potential involvement of TGF-β action in the pathogenesis of bone abnormalities in chronic kidney disease (CKD).

## 2. TGF-β Exists in Bone Tissue and Is Implicated in Bone Metabolism

In mammals, the TGF-β family consists of three isoforms: TGF-β1, TGF-β2, and TGF-β3 [11]. All TGF-β isoforms are expressed in bones, especially in the perichondrium, periosteum, and epiphyseal growth plate [12,13,14]. As in other tissues, TGF-β in osteoblasts is synthesized as a large precursor molecule called pre-pro-TGF-β, which contains the signal peptide, latency associated peptide (LAP), and C-terminal sequence. The C-terminal amino acid sequence forms the active form of TGF-β (mature TGF-β) after modifications [15,16] in the bone matrix. The latent TGF-β binding protein (LTBP) binds with latent TGF-β to form a complex, which is secreted from osteoblastic cells. Parts of this complex interact with extracellular matrix (ECM) components including fibronectin, fibrillin 1, and integrin [17,18,19,20,21]. Upon cleavage of the LAP by osteoclasts, abundant mature TGF-β is released from the latent TGF-β complex and is activated in bone resorption lacunae [22,23].

### 2.1. TGF-β Acts on Osteoblasts

The released mature TGF-β recruits perivascular mesenchymal cells to the surface of resorption lacunae and differentiates them to osteoblasts. TGF-β binds two types of TGF-β receptors: type I (RI/ALK5) and type II (RII). TGF-β RII transphosphorylates TGF-β RI in association with ALK5 and phosphorylates receptor-regulated Smads (R-Smads) including Smad 2 and 3 [11]. R-Smads 1, 5, and 8 are also partially activated by TGF-β bound to ALK1 [24,25]. R-Smads are dissociated from the tetrameric receptor complex to form complexes with Smad 4, and translocate to the nucleus to regulate transcription of target genes to induce proliferation and early differentiation of osteoprogenitor cells. TGF-β1 increases the pool of osteoprogenitors by inducing chemotaxis and proliferation mediated via Smad signaling pathways [10]. In addition, TGF-β2 induces activation of the Extracellular Signal-regulated kinase (ERK) signal that stimulates cell proliferation to enrich osteoprogenitor cells [26]. After recruiting mesenchymal stem cells, TGF-β stimulates osteoprogenitor proliferation in part by promoting the degradation of p57_kip2_, which is a cell-cycle inhibitor [27].

Smad 3 signaling has the strongest effect on the differentiation of mesenchymal stem cells to osteoblasts induced by TGF-β. In vitro study indicates that Smad 3 overexpression in cultured osteoblastic MC-3T3E1 cells enhances the bone matrix protein levels, alkaline phosphatase activity, and mineralization [28]. TGF-β-induced non-Smad-dependent pathways (noncanonical signaling pathways) are also involved in osteoblast differentiation. When TGF-β binds the TGF-β receptor complex, the phosphorylated TGF-β activation kinase 1 (TAK1) and TAK1 binding protein 1 (TAB1) complex initiates activation of the mitogen-activated protein kinase (MAPK) signaling cascade. Phosphorylated MAP kinase kinase (MKK) p38 MAPK induces type I collagen expression [29]. Previous research indicates that osteocalcin, which is an extracellularly expressed protein that interacts with hydroxyl apatite, is also regulated via TGF-β through ERK and p38 MAPK activation [30]. Bone sialoprotein, which is a major glycoprotein, is regulated by TGF-β through β-catenin signaling [31]. Recently, it has also been reported that osteoblast-derived soluble factors, including TGF-β, indeed promote metastatic potential in prostate cancer cells, and the effect by TGF-β is, at least partly, mediated by noncanonical TRAF6-dependent signaling [32]. 

Smad-dependent and -independent pathways converge at the Runt-related transcription factor 2 (Runx2) gene to control mesenchymal cell differentiation [33]. Runx2, which is also called core-binding factor alpha subunit (cbfa1), is a master regulator of osteoblast differentiation. Runx2-deficient mice completely lack osteoblasts and mineralized matrix [34]. Runx2 directly regulates the expression of several genes including type I collagen, alkaline phosphatase, osteopontin, osteonectin, and osteocalcin, because Runx2 regulates the expression of osterix, the zinc-finger containing transcription factor that regulates the nuclear factor for activated T cells (NFAT2). Cooperation of NFAT2 and osterix is required for the expression of these target genes [35,36]. TGF-β increases Runx2 expression during the early differentiation of osteoblasts, accelerating the proliferation of osteoblasts. 

The proliferated osteoblasts synthesize new extracellular matrices, including type I collagen, osteocalcin, osteopontin, alkaline phosphatase, and matrix metalloprotease-13 (MMP-13), on the bone surface [37]. In contrast, in the late differentiation stage of osteoblasts, TGF-β represses Runx2 gene expression and inhibits the terminal differentiation of osteoblasts. TGF-β-induced Smad 3 activation is involved in this pathway, which suppresses matrix mineralization via the terminal differentiation of osteoblasts [38].

Upon termination of bone matrix synthesis, osteoblasts undergo apoptosis or differentiate into osteocytes, the bone lining cells. Mature TGF-β controls osteoblast survival through blocking osteoblast apoptosis. Membrane-type MMP (MT1-MMP), which is produced by osteoblasts and activates latent TGF-β, is involved in osteoblast survival [39]. Another study revealed that the deletion of Smad 3 does not inhibit osteoblast terminal differentiation, but induces osteoblast differentiation to osteocytes. This finding indicates that Smad 3 signaling regulates the lifespan of osteoblasts and bone formation rate [40]. Therefore, TGF-β plays distinct roles at each stage of the osteoblast lifecycle.

### 2.2. TGF-β Acts on Osteoclasts

TGF-β promotes the migration of osteoclast precursors into bone, such as osteoblasts [41]. Bone marrow macrophages are osteoclast precursors, and they require the receptor activator of nuclear factor-κB ligand (RANKL) [42,43] and macrophage-colony stimulating factor (M-CSF) [44] for osteoclastogenesis. TGF-β acts directly on bone marrow macrophages and promotes osteoclastogenesis [45,46,47]. Yasui et al. [48] demonstrated that the TGF-β-induced molecular interaction between Smad 2/3 and TRAF 6 is critical for RANKL-induced osteoclastogenic signaling. In addition, RANKL and M-CSF are produced by osteoblasts. Therefore, the effects of TGF-β on osteoclasts are also derived from osteoblasts. TGF-β-stimulated osteoblasts express not only osteoblastic functional proteins including collagen type 1, alkaline phosphatase, and osteocalcin, but also osteoclast regulatory genes including M-CSF, RANKL, and osteoprotegerin (OPG) [45,49]. The effect of TGF-β on osteoclastogenesis is dose-dependent. Treatment with low-dose TGF-β enhances osteoclastogenesis by increasing M-CSF expression and prostaglandin production, as well as the RANKL to OPG ratio [50], whereas treatment with a high concentration represses M-CSF and RANKL expression while increasing OPG expression [51,52,53]. Because OPG is a high-affinity ligand for RANKL and acts as a soluble inhibitor of RANKL produced by osteoblasts, the effects of TGF-β on osteoclasts mediated by osteoblasts may serve as a negative feedback for bone remodeling.

### 2.3. TGF-β Regulates Bone Homeostasis Mediated by Osteocytes

Osteocytes are the most abundant bone cells embedded in the lacunae of the bone matrix. These cells are transformed from osteoblasts, and are speculated to function as a network of sensory cells mediating the effect of mechanical loading through the extensive lacuno-canalicular network. Because osteocytes stimulate osteoclast formation and activation through RANKL expression [54], osteocytes are able to induce and control bone remodeling.

In addition to regulating osteoclasts and osteoblasts, osteocytes also have direct resorbing capability, depositing new bone matrix surrounding the lacuno-canalicular network [55]. The purpose of this process is to maintain mineral homeostasis, the magnitude of fluid shear stress, and the mechanical properties of bone. Osteocytes drive several proteases including MMPs, cathepsin K, carbonic anhydrase 2, and tartrate-resistant acid phosphatase in perilacunar/canalicular remodeling [55,56,57,58]. TGF-β tightly regulates osteocyte MMP-13 expression. Moreover, TGF-β has been shown to be important for the stabilization of osteocytes [59]. Dying osteocytes express antiapoptotic and proapoptotic molecules, leading to the activation of osteoclast activity and bone resorption [60]. Targeted ablation of osteocytes by injection of diphtheria toxin in mice dramatically activates osteoclasts [61]. Osteocyte viability may reflect bone remodeling and integrity. TGF-β may be an important factor in maintaining bone homeostasis through regulating osteocyte function.

Given that TGF-β is an important regulator of bone remodeling and that high levels of TGF-β are found in CKD, TGF-β may be involved in bone abnormalities in CKD.

## 3. Abnormalities of Bone and Mineral Metabolism in Chronic Kidney Disease

Since the kidney plays important roles in mineral metabolism, disturbances in mineral and bone metabolism are common complications in CKD. These abnormalities were traditionally termed renal osteodystrophy (ROD), and have been renamed “Chronic Kidney Disease-Mineral and Bone Disorder (CKD-MBD)” [62].

Along with a decreasing glomerular filtration rate (GFR), serum fibroblast growth factor 23 (FGF23) and sclerostin, which are derived from osteocytes, begin to increase prior to change in parathyroid hormone (PTH) and 1,25 dihydroxyvitamin D_3_ (1,25D) concentrations. The circulating levels of these proteins increase with an accompanying decline in GFR [63,64]. FGF23 is an endocrine hormone that regulates phosphate metabolism and accelerates degradation of 1,25D in impaired phosphate excretion as seen in CKD. Hyperparathyroidism, hyperphosphatemia, and decreased circulating levels of 1,25D are observed in advanced CKD.

In CKD, bone metabolism is aggravated in response to mineral metabolism disturbance. Because PTH stimulates the speed of osteoclastic bone resorption and remodeling, an extremely high turnover of bone exists in patients with advanced CKD on stable hemodialysis or with inadequate control of PTH. Extremely high bone turnover reduces bone mineral density (BMD) and worsens the bone microstructure, leading to fragility. Bone microstructure disturbance is associated with high blood levels of PTH [65,66,67]. High turnover of bone together with high blood PTH level is often accompanied by bone marrow fibrosis. On the other hand, marked decreases in both bone resorption and bone formation caused by suppressed PTH secretion or skeletal resistance to PTH under a uremic condition are found in low-turnover bone lesions in CKD [68,69,70]. Regardless of high or low bone turnover, abnormal bone turnover increases fracture risk in CKD patients [71,72,73,74].

Ectopic cardiovascular mineralization is frequently associated with decreasing BMD and/or bone turnover disturbance. Regarding vascular calcification, the mineralization process in blood vessels shows a pattern analogous to bone mineralization, in which not only the mineralization step, but also the matrix production is regulated [75]. There are two forms of vascular calcification in CKD. One is associated with atherosclerosis, which is the intimal calcification produced by the osteoblastic transformation of cells derived from smooth muscle cells and mesenchymal cells. The other is the transformation of medial vascular smooth muscle cells to osteo/chondrocytic cells. Both types of calcifications are linked to elevated Dickkopf-1 (Dkk1), sclerostin, and activin levels [75,76]. As mentioned above, CKD-MBD is a syndrome which could result in disorders of bone metabolism and/or the cardiovascular system. On the other hand, ROD is used to indicate bone morphologic changes in patients with CKD and is one measure of the skeletal disorder component of CKD-MBD [62].

In addition to mineral disturbance, TGF-β production is associated with CKD progression [77]. Circulating TGF-β levels is a reliable biomarker of CKD [78]. In acute and chronic kidney injury, increased TGF-β levels parallel the reduced expression of bone morphogenetic protein (BMP)-7, which is also a member of the TGF-β superfamily. BMP-7 is known to be an important inhibitor of vascular calcification [79]. The reduction of BMP-7 action on the vascular wall is associated with abnormal bone turnover [79].

## 4. Possible Role of TGF-β in Chronic Kidney Disease-Mineral and Bone Disorder

### 4.1. Renal Osteodystrophy and Resistance to PTH

The possible TGF-β involvements in abnormalities of bone and mineral metabolisms in CKD patients have been reported since the 1990s. Jiang et al. [80] investigated the plasma levels of TGF-β in hemodialysis patients and found that patients with ROD have significantly higher levels of TGF-β than in patients without ROD. They concluded that the pathological condition of ROD may stimulate overproduction of TGF-β in patients undergoing hemodialysis. Using immunohistochemistry, Duarte et al. [81] also demonstrated intense TGF-β expression in bone samples with osteitis fibrosa from patients with ROD, which is accompanied by extremely high bone turnover. A study conducted by Santos et al. [82] indicated that TGF-β expression in bone changes before and after parathyroidectomy. Because TGF-β modulates the functions of both osteoblasts and osteoclasts, these results are rational. However, humoral parameters, including PTH and alkaline phosphatase, were not found to be associated with blood TGF-β concentration in Jiang et al.’s study [80]. From the above reports, it remains unclear whether the serum concentration of TGF-β reflects bone turnover. In an in situ hybridization study using bone biopsy samples, Hoyland and Picton [83] demonstrated that TGF-β signals localize predominantly to osteoblasts, although the same signals are also observed in some osteocytes and osteoclasts, and the levels decrease in renal bone samples. In that study, the level of TGF-β expression depends on bone turnover; the lowest level of TGF-β in osteoblasts is observed in adynamic bone associated with low PTH levels. This observation is consistent with in vitro results that show that PTH increases TGF-β production in cultures of normal osteoblast-like cells [84].

Liu et al. [85] reported elevated expression of TGF-β1 mRNA and its receptor, as well as TGF-β signaling in *jck* mice, a genetic model of polycystic kidney disease with progressive decline in renal function. In these mice, bone turnover increases independently of detectable PTH change. Administration of 1D11, a neutralizing anti-TGF-β antibody, to *jck* mice suppresses osteoblast and osteoclast functions and reduces bone turnover independent of renal function and changes in serum indices. These findings suggest a direct effect of 1D11 on bone rather than a systemic effect. The mechanisms of the effects of 1D11 on osteoblast and osteoclast functions are elucidated by assessing intracellular signaling pathways. The phospho-Smad 2/Smad 2 ratio in osteoblasts increases in *jck* mice, and treatment with 1D11 significantly attenuates the increase. 

Some reports reveal that there is interplay between TGF-β and Wnt signaling in osteoblasts. Using chemical kinase inhibitors, Zhou demonstrated that TGF-β stabilize β-catenin through both Smad3 and non-Smad pathways [31]. McCarthy and Centrella demonstrated that Wnt pathway induction stabilizes β-catenin and increases T cell factor/lymphoid enhance factor (TCF/LEF)-dependent gene expression in parallel with β-catenin-independent complex formation between TCF-4 and Runx2. They also presented that activation of Runx2 or TCF-4 coenhances TCF and Runx2 activity and increases TGF-β receptor I expression [86]. As described above, high TGF-β protein expression has been observed in high-turnover bones from patients with end-stage renal disease [81,82]. Existence of a positive regulatory loop between TGF-β and Wnt signaling may lead to the pathogenesis of high-turnover bone disease in CKD.

It has long been recognized that in CKD, the bone is resistant to the calcemic action of PTH. This phenomenon is seen in CKD patients [87] and also uremic rats [88,89,90]. One of the mechanisms is the downregulation of the PTH/PTH-related peptide (PTHrP) receptor mRNA in uremic rats compared to normal rats [91,92]. TGF-β attenuates PTH signaling in osteoblasts [93]. In cultured osteoblasts derived from fetal rat calvariae, TGF-β2 induces a decrease in the steady-state level of PTH/PTHrP receptor mRNA, resulting in decreased PTHrP receptor binding [93]. TGF-β binds to TGF-β receptor II (TBR2), leading to direct phosphorylation of the cytoplasmic domain of the PTH type I receptor (PTH1R). This reaction results in the formation and internalization of the TBR2 PTH1R complex [94]. Although the pathogenesis of skeletal resistance and the contributing factors remain unclear, the action of TGF-β on bone and/or osteoblasts may be involved in PTH resistance in CKD conditions such as the accumulation of uremic toxins [95,96,97]. Since PTH is a master regulator of bone turnover in CKD, these studies suggest that TGF-β may contribute to the pathogenesis of ROD.

### 4.2. High Serum FGF23 Level

As CKD progress, there is a progressive increase in serum FGF23 level, which may reach exceptionally high levels [64,98]. The blood FGF23 level is associated with increased mortality in CKD patients [99]. The regulation of FGF23 production is still incompletely understood. PTH stimulates FGF23 production [100,101]. This phenomenon is confirmed by the outcome of parathyroidectomy [100] and calcimimetic treatment [102], both of which decrease the circulating level of FGF23. Although FGF23 decreases the PTH level as a negative feedback mechanism under normal conditions [103,104], resistance to the action of FGF23 in both the parathyroid and kidney is found in advanced CKD due to downregulation of the FGF23 receptor complex, the klotho‒FGF receptor 1 (FGFR1) [105,106,107,108]. As a result, FGF23 production is maintained, resulting in the coexistence of high PTH and high FGF23 [100]. The mechanism of PTH-stimulated FGF23 production is, at least in part, mediated by activation of the orphan nuclear receptor Nurr1 [109]. On the other hand, 1,25D directly increases FGF23 transcription via vitamin D response element-mediated transcriptional activation and indirectly via extracellular signaling pathways, both of which are mediated by leptin and IL-6-induced STAT phosphorylation [110] or Nurr1 activation [109].

A recent study has found that TGF-β2 controls the production of FGF23 in UMR106 osteoblast-like cells [101]. The quantities of TGF-β-stimulated FGF23 gene and protein expression depend on the TGF-β concentration. The effect of TGF-β on FGF23 production is mediated by store-operated calcium entry (SOCE) through Orai1/STIM1 [101]. A SOCE inhibitor significantly blunts the induction of FGF23 synthesis via TGF-β2. A similar pathway is observed when activation of the inflammatory transcription factor NF-kB upregulates the Orai1/STIM1-mediated SOCE that triggers FGF23 production [111].

In uremic animals, renal FGF23 expression correlates with local TGF-β1 expression [112]. Smith et al. demonstrated that FGF23 augments FGFR4 activation and upregulation of the calcium transporter in the absence of klotho, leading to enhanced TGF-β1 autoinduction through increases of both intracellular calcium and mitochondrial reactive oxygen species [113]. Although klotho expression in bone is repressed in CKD [114], expression of FGFR4 in osteoblasts has been confirmed [115]. A feed-forward loop between TGF-β and FGF23 may exist in osteoblasts in CKD.

Moreover, reduction of 1,25D levels may contribute to the effect of TGF-β on FGF23 production in CKD. Active 1,25D inhibits downstream TGF-β signaling, due to vitamin D receptor (VDR)–phospho-Smad 3 complex formation. In CKD, reduced expression of VDR and decreased level of its ligand may contribute to hyperactive TGF-β signaling [116]. With the progression of renal dysfunction, the expression of FGF23, an important target of PTH and 1,25D, may be modulated by TGF-β, which may be involved in the pathogenesis of CKD-MBD.

### 4.3. Wnt Inhibitors

Wnt pathways increase the osteogenic commitment of bone marrow stem cells, enhance matrix formation, and decrease the apoptosis of osteoblasts and osteocytes. Wnt signaling is regulated by secreted decoy receptors (secreted frizzled-related protein, sfrp) or antagonists. Sclerostin and Dkk1 are Wnt antagonists.

In CKD patients, higher circulating sclerostin levels correlate with higher BMD and better bone microarchitecture [117,118]. Because sclerostin expression in bone increases in early CKD [119,120,121], the skeleton is speculated to be the source of increased circulating sclerostin in CKD. High sclerostin levels may reflect increased osteocyte numbers and skeletal mass [122,123]. Both serum and bone sclerostin levels correlate negatively with the histomorphometric parameters of bone turnover and osteoblastic number in dialysis patients [124,125]. In line with this observation, circulating sclerostin levels have been found to correlate negatively with biomarkers of bone formation [123,126] and resorption [123]. CKD patients with PTH within the normal range often have adynamic bone, with suppressed bone turnover. Adynamic bone or low-turnover bone may be caused by high levels of sclerostin.

PTH is known to be a regulator of SOST (the gene that encodes sclerostin9 expression) [127]. This PTH-mediated repression of SOST requires recruitment of MEF2 to a highly conserved regulatory region 35-kb downstream from the SOST gene [128]. Loots et al. [129] demonstrated the effect of TGF-β on SOST expression in UMR-106.01, a rat osteosarcoma cell line. A TGF-β isoform induces SOST expression, and a significantly higher level compared to that before induction is sustained. This induction involves Smad 3, and PTH antagonizes the TGF-β-stimulated SOST induction. Interestingly, TGF-β targets the ECR5 region, which is a distal enhancer of the SOST promoter. This enhancer that responds to PTH stimulation [128] contains a binding site for both the MEF2 family of transcription factors and Smad2/3. The above findings would suggest that TGF-β may cause repression of sclerostin expression in CKD, because high levels of PTH and TGF-β coexist in the setting of CKD. However, both serum and bone sclerostin levels are high in CKD.

On the other hand, Notsu et al. [130] reported that advanced glycation end product (AGE)-3 induces SOST expression and osteocyte apoptosis via upregulated TGF-β expression. Since AGEs accumulate in the CKD condition [131], the effect of AGE-3 on SOST expression through increased TGF-β may contribute to the high SOST level. Bone sclerostin expression is diminished in late-stage CKD compared to that in early-stage CKD [119,121]. Stage-dependent sclerostin expression may be reflected by the degree of TGF-β stimulation, because the PTH level is elevated in the late-stage, while the accumulation of AGEs occurs in all stages of CKD. Further in vivo and/or in vitro studies that assess the MEF2 transcriptional activity using models simulating CKD conditions are needed to elucidate the effect of TGF-β on sclerostin production in CKD.

Dkk1 is another Wnt inhibitor, mainly produced by osteoblasts and osteocytes [132]. In normal subjects, Dkk1 is expressed at low levels in the skin, placenta, prostate, kidney, and platelets [133]. Dkk1 expression is regulated by growth factors and hormones including calcitonin, morphogenetic proteins, PTH [134], and estrogens [135]. Although bone cells are regarded as the main producing tissue, Dkk1 expression increases during renal tubule epithelial proliferation and renal repair in early CKD [134].

Circulating Dkk1 levels are only minimally affected by gender, renal function, and age [118,136,137]. Contrary to sclerostin, the Dkk1 level does not correlate with BMD or bone histomorphometric parameters in CKD patients [125,138]. A negative correlation between blood Dkk1 level and the degree of atherosclerosis [136] or aorta calcification [137] has been observed.

Fang et al. [139] demonstrated the effect of neutralization of Dkk1 using a monoclonal antibody in ldlr^−/−^ mice. These mice have reduced GFR to an extent similar to that of patients with stage 2 CKD. Tissue levels of Dkk1 are elevated in these mice. Neutralization of Dkk1 prevents osteochondrogenic transdifferentiation of vascular smooth muscle cells, vascular calcification, and renal osteodystrophy. However, elevated Dkk1 levels and downregulation of vascular klotho (both key findings in the abovementioned mouse model) are not observed in all patients with early-stage CKD [118,140]. The effect of Dkk1 neutralization on vascular calcification remains unclear.

Circulating activin increase is accompanied by increased activin production in the kidney under CKD conditions [141]. Activin, a member of the TGF-β superfamily, increases during development [142] and in injured kidneys [143]. Agapova et al. [141] demonstrated that a ligand trap for the activin type IIA receptor (ActRIIA) significantly decreases Dkk1 expression both in the kidney and circulating levels [141]. Interestingly, the ligand trap for ActRIIA restores phospho-Smad/3 in the aorta, leading to suppressed gene and protein expression of Runx2, a transcription factor associated with osteoblast transition. The mouse model of CKD with atherosclerosis (ldlr^−/−^ mice) used by Agapova et al. [141] exhibits decreased ActRIIA expression due to overexpression of the circulating activin ligand, possibly through endocytosis and degradation. Their results suggest that ActRIIA signaling plays an important role in vascular smooth muscle cell differentiation and that high levels of Dkk1 and repression of ActRIIA signaling induce osteoblast transition from vascular smooth muscle cells without affecting serum phosphorus and PTH levels. Consequently, the activation of two factors involved in kidney development, activin and Dkk1, cooperates in CKD-induced vascular disease. The ligand trap for ActRIIA also inhibits osteoclasts to stimulate high bone turnover [144]. ActRIIA signaling not only stimulates vascular calcification and bone remodeling, but also induces left ventricular hypertrophy in an Alport syndrome model [145]. Thus, ActRIIA signaling may be the key factor that induces renal osteodystrophy, cardiovascular disease, and renal fibrosis.

## 5. Conclusions

**Figure 1:**
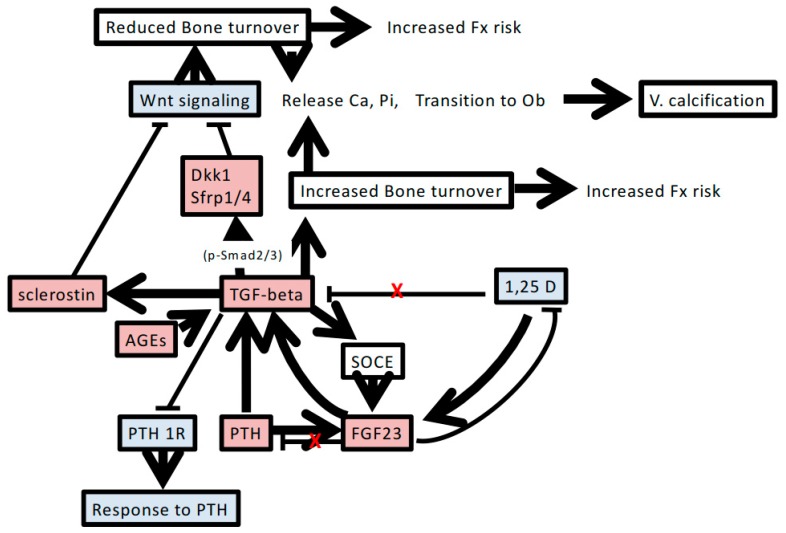
Possible factors and pathways associated with the involvement of TGF-β in chronic kidney disease-mineral and bone disorder. Increasing TGF-β levels accompanied by renal dysfunction induces mineral metabolism disturbance and bone remodeling. The detailed mechanisms and interactions are described in the text. Red-shaded boxes indicate increased levels of factors or phenomena. Blue-shaded boxes indicate decreased levels of factors. Red X indicates regulatory disturbance in chronic kidney disease. T bar indicates inhibitory action of humoral factor. FGF23, fibroblast growth factor 23; PTH, parathyroid hormone; PTH1R, PTH type 1 receptor; 1,25D, 1,25 dihydroxyvitamin D_3_; AGEs, advanced glycation end products; Dkk1, Dickkopf-1; V. calcification, vascular calcification; Ob, osteoblasts; Ca, calcium; Pi, phosphorus; SOCE, store-operated calcium entry; Fx, fracture.

In Figure 1, we summarize the possible involvement of TGF-β in abnormalities of bone and mineral metabolism in CKD. Mineral metabolism disturbance caused by renal dysfunction is inherently complicated. The involvement of TGF-β in this setting seems to increase the complexity and promote the pathogenesis of CKD-MBD. The results of a series of studies on a ligand trap activin type IIA receptor are surprising, because treatment with the ligand trap restores bone turnover as well as protects against ectopic calcification and renal fibrosis. These reports suggest that apart from activin-A, other TGF-β superfamily factors that can enhance both Smad2/3 and Wnt signaling may be involved in the pathogenesis of CKD-MBD.

On the other hand, elucidation of the involvement of FGF23 in CKD-MBD has gradually increased our understanding of the pathogenesis of CKD-MBD. A recent study shows that FGF23 increases the expression of secreted frizzled-related protein 4 and Dkk1, and these increases are not ameliorated by PTH treatment [146]. Another report shows that FGF23 also regulates local bone mineralization in a vitamin D- and klotho-independent manner [147]. Knowing the detailed interactions between FGF23 and TGF-β would facilitate better understanding of the pathogenesis of CKD as well as the search for new therapeutic targets and optimal timing of intervention.

To better understand the pathogenesis of CKD-MBD, more studies are warranted to investigate the roles of TGF-β in CKD-MBD.

## Abbreviations

Runx2	Runt-related transcription factor 2
MMP	matrix metalloprotease
RANKL	receptor activator of nuclear factor-kappa B ligand
M-CSF	macrophage-colony stimulating factor
OPG	osteoprotegerin
ROD	renal osteodystrophy
CKD-MBD	chronic kidney disease-mineral and bone disorder
GFR	glomerular filtration rate
1,25D	1,25 dihydroxyvitamin D_3_
BMD	bone mineral density
Dkk1	Dickkopf-1
